# Cardiac thrombotic stability determined by contrast-enhanced echocardiography: investigative protocol and preliminary results

**DOI:** 10.1186/s12872-021-02085-4

**Published:** 2021-05-31

**Authors:** Ying Li, Xin Wang, Weidong Ren, Yangjie Xiao, Xiaona Yu, Xueying Tan

**Affiliations:** Department of Ultrasound, Shengjing Hospital of China Medical University, No.36, Sanhao Street, Heping District, Shenyang City, 110004 China

## Abstract

**Objective:**

This study’s intent was to test a new system for scoring cardiac thrombotic stability, based on contrast-enhanced ultrasound (CEUS).

**Methods:**

We used human whole blood for an in vitro thrombotic model involving 1-h (T_1h_) and 7-day (T_7d_) subsets. The T_1h_ group was monitored for 1 h continuously to observe for the formation of a new thrombus on the original thrombus base. Changes in thrombotic CEUS images, histologic features, and shear wave elastography were recorded over time. We also studied 28 patients diagnosed with cardiac thrombi, each examined by transthoracic echocardiography and CEUS.Thrombi were scored for substrate (T_s_) and hardness (T_h_) based on the visualized degree of contrast penetration into the thrombi. Statistical analyses of T_s_ and T_h_ reflected thrombolytic time and risk of embolism to other organs.

**Results:**

Histologically, the loosely constructed ends of in vitro thrombi solidified over time. In addition, the average Young’s modulus of thrombi over time indicated a progressive increase in hardness. Contrast-enhancing agents were able to penetrate fresh, loose thrombi only, not chronic, stable thrombi. As T_s_ and T_h_ increased, prolonged thrombolytic time and greater risk of embolism to other organs were apparent.

**Conclusions:**

Our data suggest that this new CEUS scoring system correlates well with cardiac thrombotic hardness and the quality of its underlying substrate, serving to quantify thrombotic stability.

## Introduction

Cardiac thrombi formation is a frequent complication in a variety of prevalent diseases [1, 2], carrying a potential for significant morbidity and mortality from cerebrovascular and peripheral vascular events [3]. In recent years, many scholars have begun to study risk factors for thrombotic seeding. Most believe that the chronology of a thrombus formation clearly correlates with the risk of thrombotic shedding and expected thrombolytic effects [4]. In an acute stage, the unstable thrombi are less rigid, adhering loosely to vascular wall; whereas older, stable thrombi are harder, tougher, less brittle, and firmly attached to endothelial surfaces. They are not easily dislodged, and therapeutic outcomes are usually poor [5, 6]. Shear wave elastography (SWE) is a new and non-invasive imaging technique that has been broadly used (along with ultrasound) to measure tissue stiffness. It is an accurate method to determine the time of thrombosis according to the changes of thrombi hardness [7]. Therefore, the hardness of thrombi is related to the risk of falling off and the effect of thrombolysis [5]. According to the above theoretical basis, we may speculate the risk of thrombi shedding and thrombolytic effect according to the changes of the hardness of cardiac thrombi.

At present, the most commonly used method for diagnosing cardiac thrombi is TTE which often focuses on their size, number, shape, mobility, and location [8, 9]. To our knowledge, we lack the means to judge the hardness which is related to stability of cardiac thrombi at present. And the TTE image quality is suboptimal in few patients, which leads to relatively low accuracy and reproducibility [10, 11]. It has been demonstrated the introduction of a contrast agent during TTE improves image quality and display the blood supply of the tissue by observing the microbubbles of contrast medium moving with the red blood cells [12, 13]. There are only a few studies on the application of CEUS in the diagnosis of cardiac thrombosis. According to the previous study, unlike solid tumors, no contrast agent can enter the thrombi without blood supply during CEUS [11, 14, 15].

Still, it is well known that in physics, all molecules possess the physical properties of mechanical dispersion [16]. This is due to the separation and reorganization of streamline due to the interaction with the solid surface, which is affected by the pore structure of the medium under consideration [17]. Whereas contrast media fail to penetrate hard, chronic thrombi, the loosely structured ends of a soft and fresh thrombi may allow passage. If so, the mechanical diffusion properties of contrast agents offer a potential means of gauging thrombotic hardness.

We have hypothesized that contrast agents do enter the loose parts of thrombi, serving as a measure of relative hardness, and this has implications for thrombolysis.To confirm our premise, we initially examined an in vitro model of thrombosis, observing the changes and extent of thrombi formation over time and determining whether or not there is contrast agent entering fresh thrombi. We then assessed 28 patients with cardiac thrombi to further evaluate the clinical stability of such thrombosis, based on CEUS findings.

## Materials and methods

### Study participants

An in vitro model of thrombosis was established using 10.0-mL volumes of whole venous blood collected from healthy volunteers (n = 10), each drawn into a 20-mL syringe for water bath incubation at 37 °C (10 syringes in total) [18].The samples were stratified as 1-h (T_1h_) and 7-day (T_7d_) subsets (n = 5 each) and analyzed using standard two-dimensional (2-D) classical TTE, CEUS, and SWE.

From May 1, 2016 to June 29, 2019, 28 consecutive patients who had been diagnosed with intracardiac thrombi by conventional echocardiography were recruited for this study. All patient diagnoses were further confirmed clinically. This study was approved by the ethics committee of Shengjing Hospital affiliated with China Medical University, obtaining written consent in advance from all subjects. All procedures were performed in accordance with the 1964 Declaration of Helsinki and its later amendments or comparable ethical standards. Eligible patients met the following study criteria: (1) all patients have recently received thrombolysis and anticoagulation therapy (Rt-PA was used as thrombolytic agent and warfarin as anticoagulant), (2) conventional echocardiography performed at 3-day intervals, and (3) diminishing size or full resolution of cardiac masses.

### Ultrasonography

The Philip EPIC7 (with an L12-3 transducer and 3–12 MHz transmission frequency) ultrasound system was used to perform 2-dimensional classical ultrasound imaging and CEUS imaging of the thrombus in vitro. A Supersonic Aixplorer (with an L15-4 transducer and 4–15 MHz transmission frequency) ultrasound system was used to analyze the SWE of thrombus in vitro.

To examine patients with cardiac thrombosis, we used a Philips iE Elite ultrasound system (S5-1 transducer, 1–5 MHz) and QLab image analysis software (v10.8; Baltimore, MD, USA) after injecting 1–3 mL of perfluoropropane contrast as a 1% human albumin-coated microsphere formulation (Kangrun Pharmaceutical Company, Hunan, China).

### Image acquisition

T_1h_ and T_7d_ subsets of the in vitro model were studied by standard 2-D classical ultrasound, CEUS, and SWE. The T_1h_ samples were monitored continuously by ultrasound for 1 h to chronicle new thrombus formation on the original thrombus base. After 1 h continuously observe, CEUS was performed in vitro as follows: (1) normal saline was infused with contrast agent, (2) each syringe (thrombus within) was connected to this solution, and (3) the normal saline/contrast mix was slowly and continuously aspirated.SWE evaluations of thrombus proceeded as follows: (1) thrombotic contours were traced, (2) the average value of Young’s modulus (ie, minimum + maximum Young’s modulus values in kilopascal [kPa]) for the thrombus within the Q-box area (according to system) was automatically calculated, and (3) the average value of Young’s modulus (in three attempts) served for other statistical computations. We also examined T_7d_ samples by ultrasound to document chronic thrombus formation on the original thrombus base, applying the same methods above.

Patients assumed left lateral decubitus positions, recording electrocardiography (ECG) tracings simultaneously. In addition to 3–5 cardiac cycles acquired in left ventricular short-axis view (mitral valve, papillary muscle, and apical horizontal section separately), left ventricular long-axis 3-chamber, apical 2- and 4-chamber, and other nonstandard views were obtained. Thrombotic parameters were recorded as follows: location, attachment point, substrate, shape, size, echo, mobility of the thrombus and the effect of thrombus on cardiac hemodynamics. Long and short diameters of thrombi were measured at broadest segments. All patients were then examined by CEUS. Left ventricular opacification (LVO) mode was started, and contrast agent was gently mixed. Once at room temperature, 1 mL of contrast agent was injected quickly via peripheral vein. Contrast filling of the heart cavity allowed lesions to be characterized. Long and short diameters and areas of thrombi were measured at broadest segments. Entry of contrast around and at the base of each thrombus was observed and recorded. Repeated the above steps once the observation was insufficient.Finally, the patients were monitored by conventional echocardiography every 3 days. Images were stored on a hard disk for off-line analysis.

### Image analysis

For each in vitro thrombus, we recorded size, echo, activity, time-dependent changes, contrast entry, and elastography. Degrees of contrast enhancement were visually graded (1–4) in semi-quantitative analysis of cardiac thrombi, applied to substrate status (T_s_) as follows.The substrate condition of the cardiac thrombi is defined as Ts (substrate refers to the thrombus base and possible contrast penetration under the thrombus):

- *1 point* the contrast medium enters less than half of the thrombus base, and less than 3 mm in contrast medium width
- *2 points* the contrast medium enters more than half of the thrombus base, but less than 3 mm in contrast medium width
- *3 points* a wide gap (> 3 mm) of ultrasound contrast could be seen in most of the substrate
- *4 points* no or thin substrate

The hardness (T_h_) of cardiac thrombi was graded by nature of contrast penetration as follows:

- *1 point* no contrast agent was found on the surface of the thrombus
- *2 points* a small amount of dot-like contrast agent was seen on the surface of the thrombus
- *3 points* strip-like contrast agent was seen on the surface of the thrombus
- *4 points* a large amount of the contrast agent entered the surface of the thrombus

**Fig. 1:**
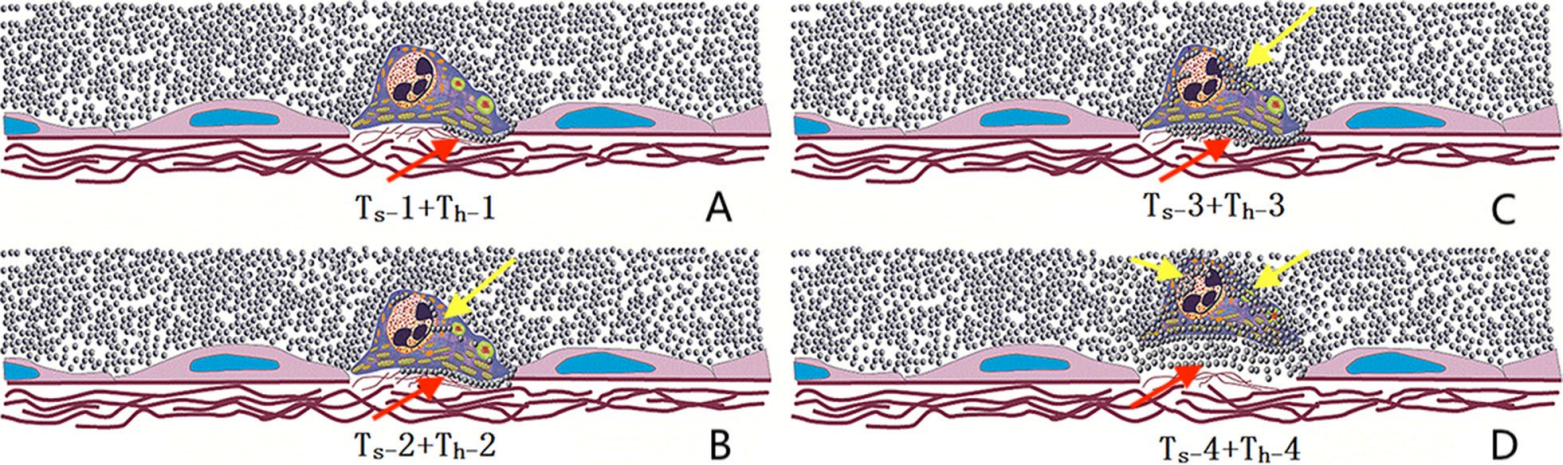
Standard application of contrast-enhanced ultrasound scoring system. **a** T_s_ 1: the contrast medium enters less than half of the thrombus base, and less than 3 mm in contrast medium width (red arrow); T_h_ 1: no contrast agent was found on the surface of the thrombus. **b** T_s_ 2: the contrast medium enters more than half of the thrombus base, but less than 3 mm in contrast medium width (red arrow); T_h_ 2: a small amount of dot-like contrast agent was seen on the surface of the thrombus (yellow arrow). **c** T_s_ 3: a wide gap (> 3 mm) of ultrasound contrast agent could be seen in most of the substrate (red arrow); T_h_ 3: strip-like contrast agent was seen on the surface of the thrombus (yellow arrow). **d** T_s_ 4: no or narrow/thin substrate (red arrow); T_h_ 4: a large amount of the contrast agent entered the surface of the thrombus (yellow arrow)

The standard CEUS scoring system is shown in Fig. 1. Total CEUS score is the sum of T_s_ and T_h_.

### Inter- and intra-observer reproducibility

To assess quantitative reproducibility, T_s_ and T_h_ were measured in all patients by two independent observers. Observers A and B (equivalent in expertise) obtained single measurements on the same day, observer A repeating the measurement after 1 week.

### Thrombolytic time and other embolic sites

All 28 patients received thrombolytic therapy and underwent conventional TTE studies at 3-day intervals until cardiac thrombi had disappeared, defined as thrombolytic time. For first-time diagnoses of cardiac thrombosis, symptoms arising elsewhere prompted ancillary exams to determine other-organ infarction. Embolism was investigated only if definitive symptoms developed, scoring the presence or absence of embolism to other organs as 1 or 0.

### Pathologic findings

In vitro thrombi were fixed in 4% formaldehyde and routinely processed, producing hematoxylin and eosin (HE)-stained sections for light microscopic examination.

### Statistical analysis

Correlations between thrombolytic time and embolism with Ts, Th, total score were analyzed by Spearman’s rho (SPSS v23.0; IBM Corp, Armonk, NY, USA), setting significance at *p* < 0.05 (two-tailed). Kappa analysis was used to test the consistency inter- and intra-observer. Kappa ≥ 0.75 showed good consistency, 0.4 ≤ kappa < 0.75 showed general consistency, Kappa < 0.4 showed poor consistency.

## Results

### Ultrasonic features of thrombi in vitro

**Fig. 2:**
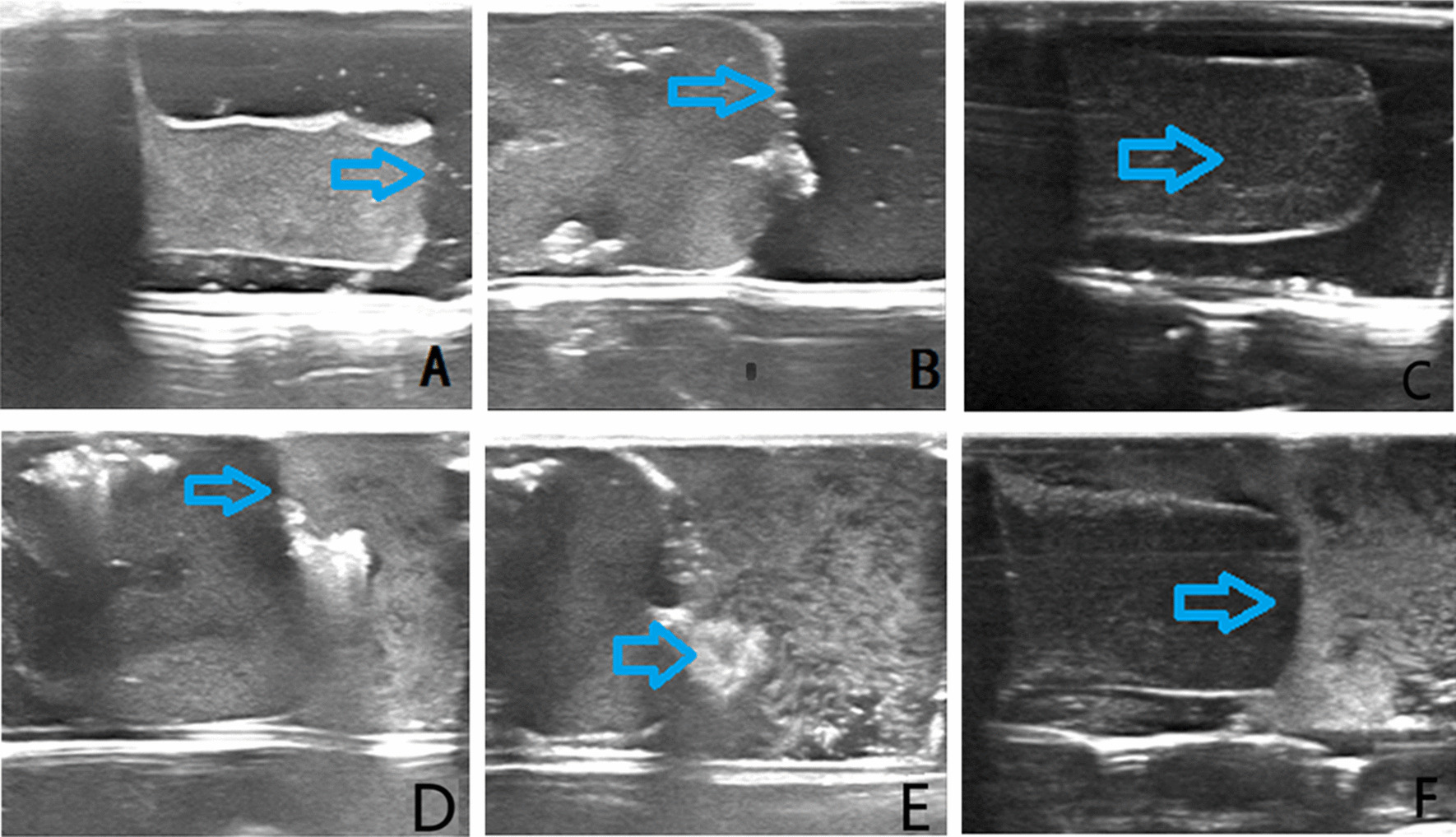
Two-dimensional conventional and contrast-enhanced ultrasound imaging of the thrombus at 1 h, 2 h and 7 days. **a** 1 h after thrombus formation, the surface of the thrombus was isoechoic (blue arrow). **b** 2 h after thrombus formation, volume increased and hyperechoic surface augmented (blue arrow). **c** 7 days after thrombus formed, uniformly hypoechoic core and slightly stronger surface echo (blue arrow). **d** 1 h after thrombus formation, ultrasound contrast enters freshly formed part of thrombus at surface (blue arrow), inconsistent with standard 2-dimensional morphology. **e** 2 h after thrombus formation, small thrombus (blue arrow) with obvious mobility on the surface of the thrombus, not shown by two-dimensional imaging. **f** No surface entry by contrast in thrombus of 7 days (blue arrow)

T_1h_ samples were observed for 1 h continuously. Thrombotic surfaces appeared isoechoic (Fig. 2a), with ultrasound contrast entering freshly formed surface areas (Fig. 2d). Contrast-enhanced morphology was thus inconsistent with traditional 2-D features. Two hours after onset of thrombosis, mean volumes (± SD) increased progressively, relative to samples at 1 h (16.32 ± 5.34 vs 28.43 ± 8.43 mL; *p* < 0.05); and thrombotic surfaces became hyperechoic (Fig. 2b). A small thrombus with obvious mobility on the surface of the thrombus was evident (Fig. 2e), although not visible by conventional 2-D imaging. In T_7d_ samples, the cores were uniformly hypoechoic, and echo at the surface was somewhat stronger by comparison (Fig. 2c). No ultrasound contrast penetrated thrombotic surfaces in this subset (Fig. 2f).

**Fig. 3:**
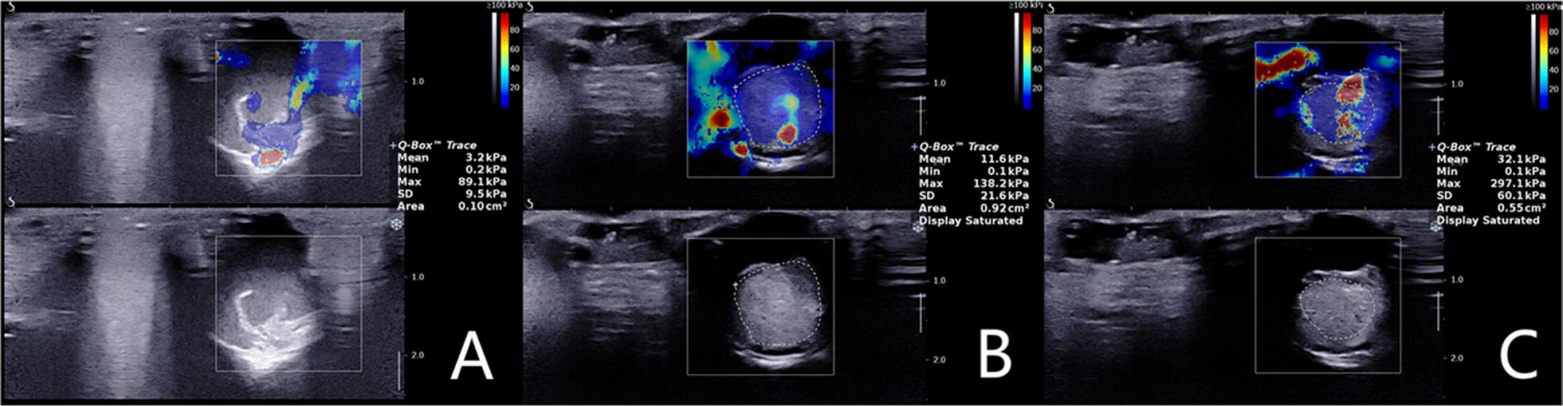
Shear wave elastography images at various time periods after thrombosis. **a** Fresh thrombosis. **b** Thrombus formed within 1 h. **c** Thrombus formed over 7 days

In SWE studies, Young's modulus (mean ± SD) was higher in T_1h_ and T_7d_ samples than in fresh thrombi, and the T_7d_ subset surpassed the T_1h_ subset in this regard (fresh, 3.85 ± 2.24 kPa; T1h, 9.85 ± 3.24 kPa; T7d, 28.58 ± 4.23 kPa; *p* < 0.05). SWE images are shown in Fig. 3.

### Pathology of thrombi in vitro

**Fig. 4:**
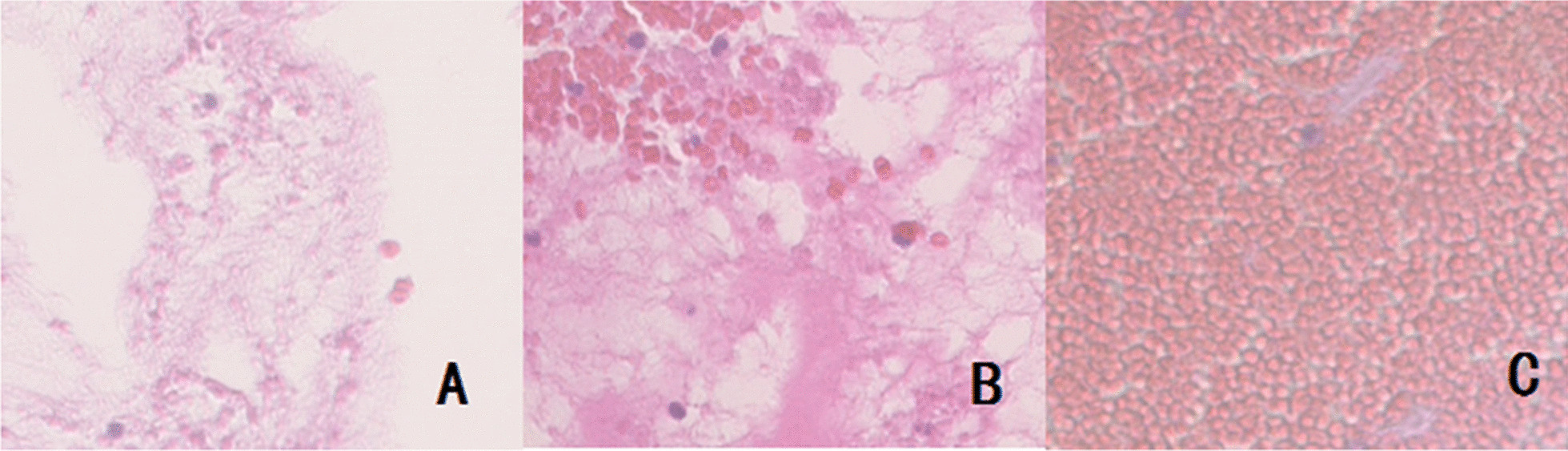
Histologic preparations of in vitro thrombus (Hematoxylin–eosin stain, 200X). **a** Loosest structure and minimal cell aggregation of newly formed thrombi. **b** Loose fibrous network and wide cell spacing 1 h after thrombus formation. **c** Uniform red blood cell and fiber network, with limited cell spacing (Day 7)

Fresh thrombi displayed the loosest structure and the least cell aggregation. After 1 h, the fiber grid was still loose, and cell spacing was sizeable. On Day 7, the red blood cell and fiber network was structurally uniform, with little intercellular space (Fig. 4).

### Population characteristics

Characteristics of the patient population are summarized in Table 1. Mean patient age was 56.6 ± 17.2 years, and no adverse drug reactions occurred. Significantly more men were affected (17 vs 11; *p* < 0.05). Among the 28 patients recruited, 20 (71.43%) had one type of cardiac disease: 4 (14.29%) coronary heart disease, 4 (14.29%) atrial fibrillation, 4 (14.29%) hypertension, 7 (25%) cardiomyopathy and 1 (3.57%) endocardial fibroelastosis. Six patients (21.43%) had two or more types of cardiac disease. Two patients (7.14%) denied heart disease history. However, after admission, one was found heart failure and the other one was diagnosed deep vein thrombosis in lower extremities.Thrombolytic times ranged from 3 to 90 days. Ten patients (35.71%) exhibited other embolic sites, including brain, kidney, abdominal aorta, and posterior tibial artery.

**Table 1:** Descriptive characteristics of all patients

N	Age	Sex	History	Position	Diameter (mm)	T_s_	T_h_	Total score	Thrombolysis time (days)	Embolism in other sites
1	49	F	Rheumatic valvular heart disease**,** Atrial fibrillation	Left atrium	45 × 33	1	2	3	18	Brain
2	75	M	Atrial fibrillation	Left atrial appendage	40 × 22	1	1	2	21	None
				Left atrial appendage	16 × 10	1	1	2	18	None
3	66	F	Hypertension, Heart failure	Left ventricle	43 × 22	1	1	2	90	None
4	24	M	Heart failure	Left ventricle	31 × 14	2	2	4	18	Brain
5	31	F	Deep vein thrombosis in lower extremities	Right ventricle	37 × 27	4	4	8	6	Pulmonary artery
6	12	F	Endocardial fibroelastosis, Heart failure	Left ventricle	51 × 25	3	4	7	18	Brain,Kidney,Abdominal aorta
				Left ventricle	37 × 29	4	4	8	6	Brain,Kidney,Abdominal aorta
				Left ventricle	10 × 10	4	4	8	3	Brain,Kidney,Abdominal aorta
7	34	M	Rheumatic valvular heart disease**,** Atrial fibrillation	Left atrium	34 × 25	1	1	2	60	None
8	37	M	Rheumatic valvular heart disease**,** Atrial fibrillation	Left atrium	49 × 25	2	2	4	21	None
				Left atrium	35 × 29	2	3	5	21	None
9	56	M	Atrial fibrillation**,** Coronary heart disease	Left atrium	52 × 45	3	3	6	27	Brain
				Left atrium	43 × 37	2	3	5	24	None
10	55	M	Rheumatic valvular heart disease, Atrial fibrillation**,** Hypertension	Left atrium	41 × 25	2	2	4	18	Spleen
11	67	F	Atrial fibrillation**,** Hypertension, Heart failure	Left ventricle	39 × 26	2	1	3	18	None
12	81	M	Atrial fibrillation	Left atrium	22 × 18	2	3	5	24	None
13	62	M	Atrial fibrillation	Left atrium	51 × 34	4	4	8	6	Brain,kidney
14	59	M	Atrial fibrillation	Left atrium	52 × 23	4	3	7	9	None
15	63	M	Hypertension, Heart failure	Left ventricle	39 × 33	1	1	2	45	None
16	69	M	Coronary heart disease, Heart failure	Left ventricle	42 × 37	2	2	4	24	None
17	72	M	Hypertension, Heart failure	Left ventricle	47 × 29	3	3	6	12	None
18	74	M	Coronary heart disease, Heart failure	Left ventricle	45 × 36	2	2	4	21	Posterior tibial artery
19	45	M	Coronary heart disease, Heart failure	Left ventricle	44 × 38	1	1	2	27	None
20	45	M	Coronary heart disease, Heart failure	Left ventricle	39 × 29	2	2	4	24	None
21	49	M	Hypertension, Heart failure	Left ventricle	48 × 45	2	2	4	24	Brain
				Left ventricle	22 × 21	2	3	5	21	Brain
22	53	M	Dilated cardiomyopathy, Heart failure	Left ventricle	53 × 43	2	3	5	24	Brain
				Left ventricle	34 × 21	2	3	5	24	Brain
23	55	F	Dilated cardiomyopathy, Heart failure	Left ventricle	52 × 23	1	2	3	24	None
24	63	F	Dilated cardiomyopathy, Heart failure	Left ventricle	45 × 29	2	1	3	21	None
25	67	F	Dilated cardiomyopathy, Heart failure	Left ventricle	37 × 29	1	1	2	36	None
26	68	F	Dilated cardiomyopathy, Heart failure	Left ventricle	39 × 34	2	2	4	21	None
27	78	F	Dilated cardiomyopathy, Heart failure	Left ventricle	43 × 22	1	1	2	39	None
28	76	F	Dilated cardiomyopathy, Heart failure	Left ventricle	34 × 26	2	3	5	24	None

### Echocardiographic features of cardiac thrombi

Most patients (22/28, 78.57%) showed single-site involvement, only a few demonstrating two or more concurrently involved sites (multiple: 6/28, 21.43%; two: 5/28, 17.85%; three: 1/28, 3.57%)i. A total of 35 thrombi were identified. There were two thrombi of left atrial appendage (5.71%), 10 of left atrium (28.57%), 22 of left ventricle (62.86%), and one of right ventricle (2.86%).

By standard TTE, cardiac thrombi presented as mass-like echoes of the heart, with irregular, round, crescenteric, or wedge shapes. Most had wide bases, and a few had no bases at all. They were largely devoid of mobility or deformation. Those thrombi without bases were connected to the heart by slender pedicles, showing obvious mobility and slight deformation. Echoes of these thrombi were either hypoechoic, hyperechoic, or mixed. Thrombi size ranged from 16 × 10 mm to 53 × 43 mm.

CEUS confirmed that contrast might penetrate the surface and base in a portion of thrombi. The contrast images of some thrombi were inconsistent with traditional 2-D images. We ranked contrast images (Table 1) using a defined contrast-enhanced scoring system.

**Fig. 5:**
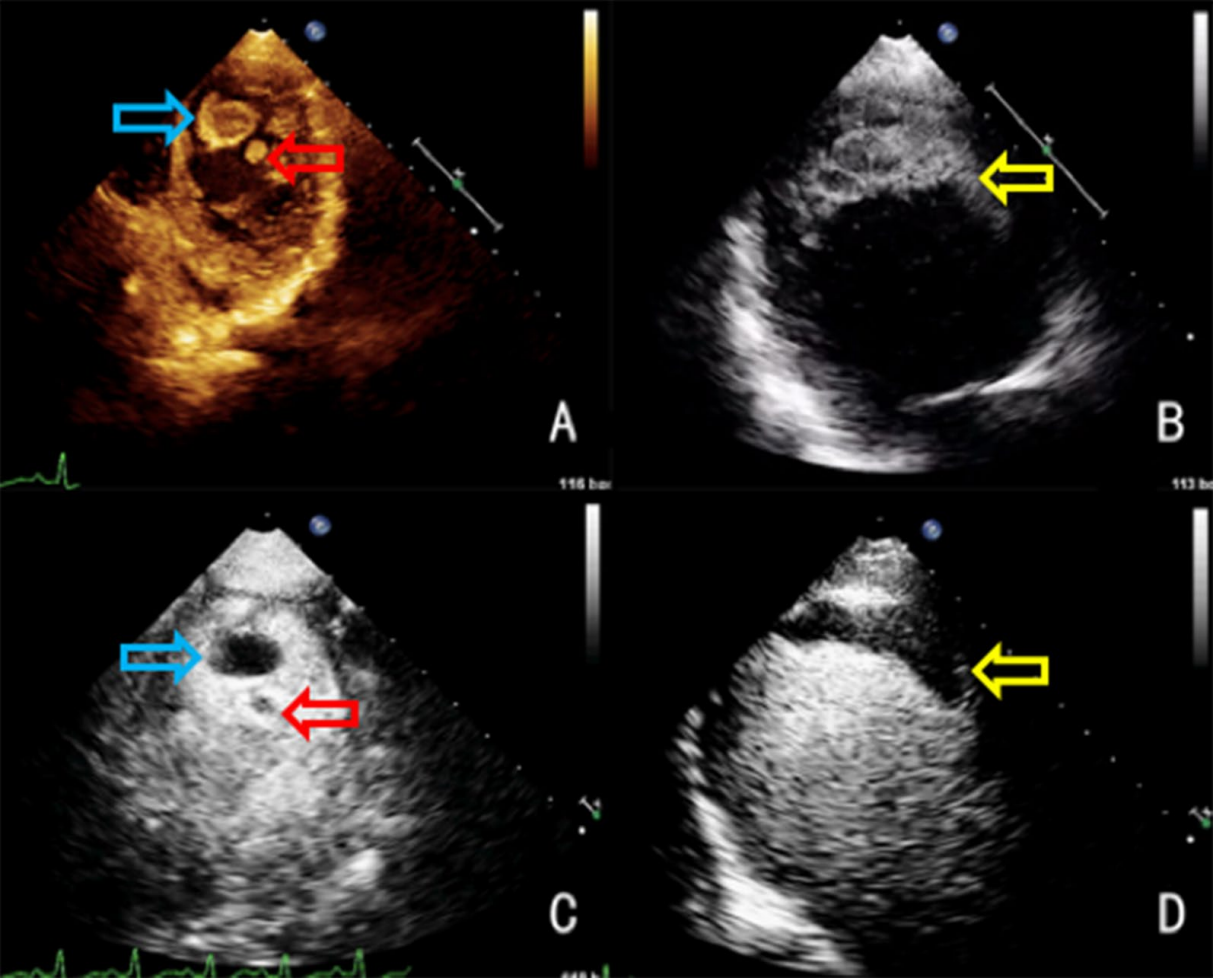
Female child (12 years of age) with endocardial fibroelastosis for 11 years, diagnosed with cerebral, renal, and lower extremity arterial embolism (TTE and CEUS showing three thrombus of left ventricular cavity, all resolved after thrombolytic therapy). **a** TTE of two solid, quasi-circular thrombi of left ventricle (thrombus 1, blue arrow; thrombus 2, red arrow). **b** TTE of one solid, irregular thrombus at apex of left ventricle (thrombus 3, yellow arrow). **c** CEUS showing voluminous contrast entering thrombus 1 (blue arrow) and thrombus 2 (red arrow), inconsistent with standard 2-dimensional morphology (no substrate for either, CEUS score [T_s_ + T_h_] of 4 + 4). **d** CEUS showing voluminous contrast around thrombus 3 (yellow arrow), wide gap filled with contrast across most of base (CEUS score of 3 + 4)

In a representative case, we have shown the CEUS score of three thrombi. A 12-year-old girl with endocardial fibroelastosis of 11 years’ duration was diagnosed with cerebral, renal, and lower extremity arterial embolism. TTE revealed three thrombi within left ventricular cavity (Fig. 5a, b), and CEUS verified the contrast agent entered the surface of the three thrombi. The morphology was inconsistent with traditional 2-D features. No substrate was apparent in thrombus 1 or 2 (Fig. 5c). The total CEUS score (T_s_ + T_h_) was 8 (4 + 4). Both thrombi (1 and 2) disappeared within 6 days of thrombolytic therapy. A wide gap allowed accumulation of contrast across most of the base in thrombus 3 (Fig. 5d). The total CEUS score was 7 (3 + 4). This thrombus resolved 18 days after thrombolytic therapy.

### Relations between thrombus CEUS score, thrombolytic time, and embolism to organs other than heart

Ultimately, T_s_, T_h_ and total score correlated with thrombolytic time, increases in these values prolonging the time for thrombolysis (Table 2). Our results also suggested that T_s_, T_h_, and total score correlate with embolism to other organs, again heightened risk associated with higher values (Table 2).

**Table 2:** Associations between the new proposed CEUS parameters and thrombolysis time, and embolism in other sites

Factors	Thrombolysis time		Embolism in other sites	
	*r*	*p*	*r*	*p*
T_s_	− .636	0.000*	.561	0.000*
T_h_	− .499	0.002*	.555	0.001*
Total score	− .557	0.001*	.561	0.000*

* indicates *p* < 0.05

### Inter- and intra-observer reproducibility

Kappa value of inter-observer variabilities in Ts and Th measurements were 0.782 (*p* < 0.05) and 0.765 (*p* < 0.05), respectively. Kappa value of intra-observer variabilities were 0.786 (*p* < 0.05) and 0.767 (*p* < 0.05), respectively.

## Discussion

Cardioembolic events account for up to 30% of all ischemic strokes [2]. Accurate gauging of thrombotic stability is critical, helping to guide therapy and procedural planning [19]. TTE is widely accepted as the primary screening tool in this setting, because it is noninvasive, convenient, and usually highly specific and sensitive [9]. However, there are many limitations [10]. First of all, the accuracy of TTE in detecting thrombi is undermined if image quality is poor. More importantly, TTE fails to provide any in-depth information on thrombotic stability. CEUS is a technology developed since the mid-1980s that offers improved image quality and delineates tissue blood supply as contrast microbubbles circulate with red blood cells [20]. Consequently, we chose to examine cardiac thrombi by CEUS, devising an adjunctive scoring system for the first time. According to a previous study, thrombi (unlike solid tumors) do not permit penetration of contrast agent during CEUS in the absence of blood supply. However, this prior investigation has ignored their unique and time-dependent properties [21]. We have wondered if a loosely structured fresh thrombi resists contrast agent entering, similar to an organized thrombi.

Our research encompassed both an in vitro study and a subsequent clinical trial in humans, to include CEUS imaging of 28 patients with cardiac thrombi. In the first part, we observed morphologic and SWE changes occurring in thrombi over time in vitro experiments, documenting the contrast medium entering by chronologic phase. This was done to determine whether or not contrast agent entering is a function of thrombotic hardness.

SWE permits quantitative evaluation of tissue hardness in a noninvasive manner by way of Young’s modulus, an objective measure of elasticity [22]. The higher the Young’s modulus is, the harder the tissue [23]. We used real-time quantitative SWE to determine changes in thrombi hardness, measuring elastic values of in *v*itro thrombi at various points in time. Our data indicate that compared with fresh thrombi, values of Young’s modulus increase as thrombi mature, the T_7d_ subset surpassing the T_1h_ subset in this respect. Through SWE, we found that thrombotic hardness and stability are related and that the risk of thrombotic fragmentation and shedding increases as time goes by. We also determined that during thrombus formation, cell aggregation increases internally, achieving greater density in histologic sections. Based on pathologic findings, we have further confirmed that the porosity of thrombus diminishes over time. Thus, as porosity declines, thrombotic harness increases.

As our continuous 1-h monitoring of thrombi has proven, contrast does penetrate a freshly formed thrombi through large, loosely structured ends. This is a common phenomenon that was not observed in T_7d_ samples. Contrast agent that does enter a space-occupying area is not only reflecting the level of angiogenesis, but also the loose ends structure associated with hardness, providing a basis for gauging thrombotic stability. Although it is difficult to distinguish the initial stage of thrombosis by conventional 2-D ultrasound, given the low and weak echo, a correct diagnosis is feasible by CEUS.

In the second part of our study, we sought to verify the correlation between contrast entry and thrombotic stability.We also discovered that the extent of contrast penetration parallels the status of thrombotic substrate, and we have devised a scoring system combining contrast entry and substrate conditions to systematically rank CEUS images of cardiac thrombi. To determine the clinical ramifications of this new scoring system in measuring thrombotic stability, we analyzed T_s_ and T_h_ values, thrombolytic times, and embolism to other organs, preliminarily concluding that higher T_s_, T_h_, and total scores contribute to prolonged thrombolytic times and heighten the risk of other organ embolism. The higher the T_s_ score, the more unstable the base is; and the higher the T_h_ score, the looser the thrombi, imparting greater risk of local shedding but improved thrombolytic effect. Degrees of T_s_ and T_h_ reflect the stability of cardiac thrombi and are associated with thrombotic hardness.

In summary, a loosely structured fresh thrombus is not averse to contrast agent entering, unlike denser, mature thrombus. This was proven by our initial experimentation. However, our contrast coating is albumin, which may present different characteristics aside from those with lipid coating. This may explain why it penetrates thrombus. The second and clinically oriented investigation confirmed that the degree of contrast penetration equates with thrombotic stability, thereby indicating the prognosis of cardiac thrombi. Ultimately, it may be possible to predict patient prognosis, formulate therapeutic plans, and manage clinical care using our novel CEUS scoring system.

### Limitations

Due to cost and time constraints, CEUS is not widely usable in clinical practice. As a result, we have not amassed large volumes of CEUS data on cardiac thrombi. Besides, in vitro thrombi cannot completely simulate in vivo pathologic changes. Furthermore, based alone on symptoms we may miss some embolic events. Going forward, we intend to further expand patient sampling for in vivo studies to accrue more information.

## Conclusions

CEUS may permit detection of newer thrombi that are difficult to identify by conventional TTE. Our CEUS scoring system is also promising, helping to predict thrombolytic times and the potential for embolism to other organs by ranking thrombotic hardness and substrate status. It may prove useful for determining thrombotic stability in subsequent studies.

## Data Availability

The datasets used in the manuscript are available from the corresponding author upon reasonable request.

## Abbreviations

CEUS: Contrast-enhanced ultrasound
SWE: Shear wave elastography
T_h_: Thrombus hardness
T_s_: Thrombus substrate
TTE: Transthoracic echocardiography

## References

[1] Mollazadeh R, Ostovan MA, Abdi Ardekani AR (2009). Right cardiac thrombus in transit among patients with pulmonary thromboemboli. Clin Cardiol.

[2] Hannon N, Sheehan O, Kelly L (2010). Stroke associated with atrial fibrillation—incidence and early outcomes in the North Dublin Population Stroke Study. Cerebrovasc Dis.

[3] Sen S, Lima JAC, Oppenheimer SM (2004). Changes in cardiac thrombus status after cerebral ischemia. Cerebrovasc Dis.

[4] Rubin JM, Xie H, Kim K (2006). Sonographic elasticity imaging of acute and chronic deep venous thrombosis in humans. J Ultrasound Med.

[5] Yi X, Ni C, Li M, Wu Q, Li Y (2017). Correlation between biological characteristics of thrombus and short-term efficiency of thrombolytic therapy by acoustic radiation force impulse imaging technique. Int Angiol.

[6] Takimura H, Hirano K, Muramatsu T (2014). Vascular elastography: a novel method to characterize occluded lower limb arteries prior to endovascular therapy. J Endovasc Ther.

[7] Sigrist R, Joy L, ElK A (2017). Ultrasound elastography: review of techniques and clinical applications. Theranostics.

[8] Weinsaft JW, Kim HW, Crowley AL (2011). LV thrombus detection by routine echocardiography insights into performance characteristics using delayed enhancement CMR. JACC-Cardiovasc Imaging.

[9] Whalen H, Dako F, Patel P (2019). Role of imaging for suspected cardiac thrombus. Curr Treat Options Cardiovasc Med.

[10] Groeneveld NS, Guglielmi V, Leeflang MMG (2020). CT angiography vs echocardiography for detection of cardiac thrombi in ischemic stroke: a systematic review and meta-analysis. J Neurol.

[11] Mansencal N, Revault-d'Allonnes L, Pelage J-P, Farcot J-C, Lacombe P, Dubourg O (2009). Usefulness of contrast echocardiography for assessment of intracardiac masses. Arch Cardiovasc Dis.

[12] Pitre-Champagnat S, Leguerney I, Bosq J (2015). Dynamic contrast-enhanced ultrasound parametric maps to evaluate intratumoral vascularization. Investig Radiol.

[13] Strachinaru M, Damry N, Duttmann R (2016). Ultrasound contrast quantification for the diagnosis of intracardiac masses. JACC Cardiovasc Imaging.

[14] Zhang XT, Li Y, Ren SH (2019). Isolated metastasis of hepatocellular carcinoma in the right ventricle. BMC Cardiovasc Disord.

[15] Wang X, Li Y, Ren W, Yu X, Tan X (2020). Clinical diagnostic value of contrast-enhanced ultrasonography in the diagnosis of cardiac masses: a pilot study. Echocardiography.

[16] Grant GP, Gerhard JI. Simulating the dissolution of a complex dense nonaqueous phase liquid source zone: 2. Experimental validation of an interfacial area - based mass transfer model. Water Resour Res. 2007;43(12):W12410.

[17] Wang H, Lun Z, Lv C (2017). Measurement and visualization of tight rock exposed to CO2 using NMR relaxometry and MRI. Sci Rep.

[18] Bader KB, Gruber MJ, Holland CK (2015). Shaken and stirred: mechanisms of ultrasound-enhanced thrombolysis. Ultrasound Med Biol.

[19] Li T, Peng R, Wang F (2020). Lysophosphatidic acid promotes thrombus stability by inducing rapid formation of neutrophil extracellular traps: a new mechanism of thrombosis. J Thromb Haemost.

[20] Senior R, Becher H, Monaghan M (2017). Clinical practice of contrast echocardiography: recommendation by the European Association of Cardiovascular Imaging (EACVI) 2017. Eur Heart J Cardiovasc Imaging.

[21] Morris TA (2011). Natural history of venous thromboembolism. Crit Care Clin.

[22] Xue E, Yu Y, Lin L, Li Z, Su H (2020). Application value of real-time shear wave elastography in differential diagnosis of testicular torsion. Med Ultrason.

[23] Xiaona L, Na L, Chaoy W (2017). Effect of pathological heterogeneity on shear wave elasticity imaging in the staging of deep venous thrombosis. PLoS ONE.

